# Real-time ultrasound to assess the umbilical catheter position in neonates: a randomized, controlled trial

**DOI:** 10.1038/s41372-024-02128-6

**Published:** 2024-10-09

**Authors:** Lalita Ponin, Chayatat Ruangkit, Nichanan Ruangwattanapaisarn, Pracha Nuntnarumit

**Affiliations:** 1 Department of Pediatrics, Faculty of Medicine Ramathibodi Hospital, Mahidol University, Bangkok, Thailand; 2 Ramathibodi Medical School, Chakri Naruebodindra Medical Institute, Faculty of Medicine Ramathibodi Hospital, Mahidol University, Samut Prakan, Thailand; 3 Department of Diagnostic and Therapeutic Radiology, Faculty of Medicine Ramathibodi Hospital, Mahidol University, Bangkok, Thailand

**Keywords:** Outcomes research, Developing world

## Abstract

**Objective:**

To compare real-time ultrasound (RT-US) use as an adjunct tool to verify umbilical catheter placement versus standard care without ultrasound.

**Study design:**

Neonates requiring umbilical venous catheter (UVC) and umbilical artery catheter (UAC) placement were randomized into the standard formula (No-US) and the RT-US groups. X-rays were used to confirm the catheter position.

**Result:**

Fifty and forty-nine neonates were in the RT-US and No-US groups, respectively. RT- US showed a significantly higher rate of initial X-ray-confirmed proper catheter position than No-US (*p* < 0.001). The rates of proper positions of UVCs and UACs were significantly higher in the RT-US group than in the No-US group (both *p* < 0.001). Neonates in the RT-US group required fewer catheter adjustments and subsequent X-rays than those in the No-US group.

**Conclusion:**

RT-US enhances the accuracy of UVC and UAC placement, reduces catheter adjustments, and the number of X-rays required.

**Trial registration:**

TCTR20190622001

## Introduction

Umbilical catheterization is one of the most frequently used central intravascular accesses in the neonatal intensive care unit (NICU). The umbilical venous catheter (UVC) is commonly used for medication and intravenous fluid infusion, while the umbilical arterial catheter (UAC) is used for blood sampling and blood pressure monitoring [1]. The proper placement of this central catheter access is critical. Malposition of UVCs and UACs may cause thrombosis, organ ischemia, arrhythmia, and fluid extravasation into the pericardial, pleural, peritoneal cavity, or liver [2].

The usual practice for confirming the umbilical catheter tip position in the neonatal NICU is using a predefined formula to calculate the length of catheter insertion and confirm the tip position by conventional radiography [3, 4]. Although the modern mobile-digital X-ray imaging station provides instant bedside imaging, this technology may only be available in some hospitals, and the time to acquire the image can be lengthy and varied. In the last decade, ultrasound (US) has increasingly become a vital bedside tool in the NICU. The utilization of US as a point-of-care instrument for evaluating the structure and function of neonatal organs provides vital, bedside information in real-time for newborn care [5]. Increasing evidence from observational studies and randomized, controlled trials (RCT) has suggested that US can be used to assess the tip of the central line in neonates to verify the proper placement position [6, 7]. There are advantages of using US as an additional tool or replacing an X-ray to confirm the tip of the central line. These advantages include increased accuracy of line placement, a decreased time to obtain confirmation of catheter placement, decreased radiation exposure, and decreased imaging cost [8, 9]. Despite the widespread use of US, evidence from RCT supporting the use and benefits of US as a point-of-care tool to assess umbilical catheter (UVC and UAC) placement in neonates is scarce [10–12]. Therefore, we performed an RCT to compare US use as an adjunct tool to help verify umbilical catheter placement with the standard of care without using US. We hypothesized that using US to help confirm the umbilical catheter tip at the bedside would increase the accuracy of catheter placement and decrease the requirement for catheter manipulation, repeated X-ray confirmation, the time to perform the procedure, and complications.

## Materials and methods

This open-label RCT was conducted in the three NICUs affiliated with the Faculty of Medicine Ramathibodi Hospital, Mahidol University, Thailand. Clinical study rules and regulations were followed at each facility. The study was approved by the Ethics Committee of the Faculty of Medicine, Ramathibodi Hospital (ID: MURA2023/26). The recruitment of patients commenced on 17 January 2023 from the two NICUs located at the Faculty of Medicine Ramathibodi Hospital main campus, which included the main building NICU (RamaPYT) and Somdech Phra Deparatana Medical Center building NICU (SDMC), Phayathai, Bangkok. The enrollment of patients was slow. Therefore, an addendum was issued to incorporate the NICU at Chakri Naruebodindra Medical Institute, Bang Pla, Samut Prakan (CNMI), effective from 4 July 2023. The recruitment ended on 31 January 2024, as adequate patient numbers in both groups were reached. The study was also registered in the Thai Clinical Trials Registry (TCTR20230906002). Informed consent was obtained from the patients’ parents before enrollment.

Neonates were eligible for the study if they required UVC or UAC insertion as part of standard clinical care on admission. We excluded neonates with major structural heart disease, anomalies of the chest wall and abdomen, or unstable vital signs before enrollment. After informed consent was obtained, the neonates were randomly assigned to one of the two following groups. The control group was called the non-ultrasound (No-US) group in which catheter placement was estimated using a standard formula and the catheter position was confirmed by X-ray. The intervention group was called the the real-time ultrasound (RT-US) group in which catheter placement was guided by US and confirmed by X-ray. Randomized allocation was performed using opaque, sealed, sequentially numbered envelopes. Each envelope contained a computer-generated, block of four stratified randomization by birth weight as follows: <750 g, 750–1250 g, and >1250 g. Each NICU participated in the study had their own stratified, randomization assignments list. An enrollment log was kept to ensure that all envelopes were accounted for and used in the correct order. Blinding of the clinicians was impossible because of the nature of the intervention. The radiologist (NR) who reviewed the images (X-ray and US) retrospectively was unaware of the group allocation.

The decision to perform umbilical catheter insertion depended on the patient’s condition and the physician’s discretion. Argyle™ single-lumen, polyurethane, umbilical vessel catheters (Cardinal Health, Inc., Dublin, OH) were used in all three centers participating in the study. A 3.5 Fr catheter was used for the UAC, and a 5.0 Fr catheter was used for the UVC. Umbilical catheters were placed by pediatric residents, neonatal fellows, or neonatologists under sterile conditions. In the No-US group, the UVC and UAC were inserted to a depth that was calculated according to Shukla’s formula: UVC (cm) = [3 × weight (kg) + 9]/2 + 1 and UAC (cm) = [3 × weight (kg) + 9] (with addition of the umbilical stump length for individual patients) [13].

In the RT-US group, RT-US was performed directly during umbilical catheter insertion by one of nine physicians, who were trained to perform US for catheter tip assessment, using a sterile technique. The US team included eight neonatal fellows in training and a NICU staff member (CR). An RT-US training course for neonatal fellows was provided to help them assess the umbilical catheter tip. This course consisted of 6 h of formal demonstration and hands-on training by a pediatric radiologist (NR) and a neonatologist who was trained in neonatal US (CR). All fellows had to be approved by pediatric radiologist (NR) before performing the study. The tip of the UVC was identified by the subxiphoid right parasagittal view. The US probe was placed longitudinally at the right subcostal area to visualize the catheter’s tip at the inferior vena cava to right atrial junction. Further confirmation in the parasternal short axis, apical four-chamber, and other windows was performed as required. The subxiphoid transverse and longitudinal views were used for visualizing the UAC tip. The proper position of catheters in US was defined as being in the thoracic aorta, above the diaphragm by 1 cm for UACs, and at the inferior vena cava to right atrial junction, outside of the liver, for UVCs [14]. The US machines used were as follows. A Toshiba Xario 100 Platinum US Scanner with a cardiac 4–12 MHz 10S4 Probe (Canon Medical System, Otawara, Japan) was used in RamaPYT. A Sonosite Edge II portable US machine with a 4–8 MHz P10x phased array sector US probe transducer with a cardiac probe (Fujifilm SonoSite, Inc., Bothell, WA) was used in CNMI. A GE Vivid IQ machine with a cardiac 12S-RS Probe, Sector, 4–12 MHz (GE Healthcare, Milwaukee, WI) was used in SDMC. Finally, using a mobile digital X-ray imaging station, we performed an anteroposterior X-ray of the neonate’s chest and abdomen, taken immediately after insertion to determine the catheter tip location. This X-ray was used as the gold standard for catheter tip assessment in both groups. Lateral X-ray were not regularly performed in our units. All US and X-ray images were recorded and retrospectively reviewed by a pediatric radiologist (NR).

The collected data included neonates’ demographic data and procedural information. The procedural information comprised the indication for insertion, the date and time of the procedure, the physician who performed the procedure, umbilical catheter size and type, the number of umbilical catheters placed, the desired and final umbilical catheter depth, the umbilical catheter tip position on an US examination and on an X-ray, the duration of catheter insertion, the duration of US performance, the X-ray waiting time, the total duration of the procedure, US operator, the total number of X-rays performed, and patient’s complications during the procedure.

The primary outcome was the rate of the proper position of the umbilical catheter tip in the No-US and RT-US groups at the first X-ray after complete umbilical catheter(s) insertion. The proper catheter tip position in the X-ray was defined as when the catheter tip was at the level of the diaphragm ±0.5 cm for the UVC, and at the level of the sixth to ninth thoracic vertebrae for the UAC [1]. Any other position of the umbilical catheter than those listed above was considered improper. The secondary outcomes included the following: the number of X-rays, umbilical catheter adjustments, catheter removals, and catheters in an improper position (but acceptable) (e.g., a UVC below the liver’s edge or a UAC at the L3-L4 level); the radiation exposure dose; the cost of imaging; the duration of the US performance, X-ray waiting time, and total procedure time; and complications related to catheter insertion (e.g., hypothermia, hypoxia, hypotension, brady-tachycardia, and central line infection/sepsis).

### Statistical analysis

A univariate analysis was performed to identify significant differences between the groups. Student’s *t* test was used for comparison of parametric continuous variables, and the Mann–Whitney U test was used for comparison of non-parametric continuous variables. Pearson’s chi-square test or Fisher’s exact test was used to compare categorical variables. The Poisson regression model was used for discrete count data variables, and the results are presented as the number of cases and relative risk and confidence interval (CI). Cohen’s k was used to evaluate the inter-rater reliability between two US operators for categorical variables. A *p* value < 0.05 was considered statistically significant. Statistical analyses were performed using Stata version 18.0 (Stata, College Station, TX).

### Sample size calculation

A power calculation was used to calculate the sample size required to evaluate the accuracy of line placement. Our unit typically uses the Shukla’s formula to determine the UVC and UAC lengths [13]. Previous studies have reported that the accuracy of this formula ranges from 17.8% to 90.6% [9, 11, 15–19]. On the basis of our experience, we found that, approximately 50% of the time, the first X-ray showed the correct catheter position without the requirement for additional adjustments. We hypothesized that the RT-US procedure to visualize the catheter tip would increase the accuracy of the appropriate line placement in the first X-ray up to 75% of the time. According to this assumption, 62 umbilical catheters in each group were required to detect a post-intervention difference with 80% power and two-sided alpha of 0.05. To allow for attrition, an additional 20% or 13 umbilical catheters were added to each group. Therefore, 75 umbilical catheter insertions were required in each group. If a UAC and UVC were placed in all enrolled neonates in the study, only 38 neonates in each group would be required. However, because some critically ill neonates might receive only one umbilical catheter (UVC or UAC), we estimated that 50 neonates in each group would be required to reach the sample size goal.

## Results

**Fig. 1:**
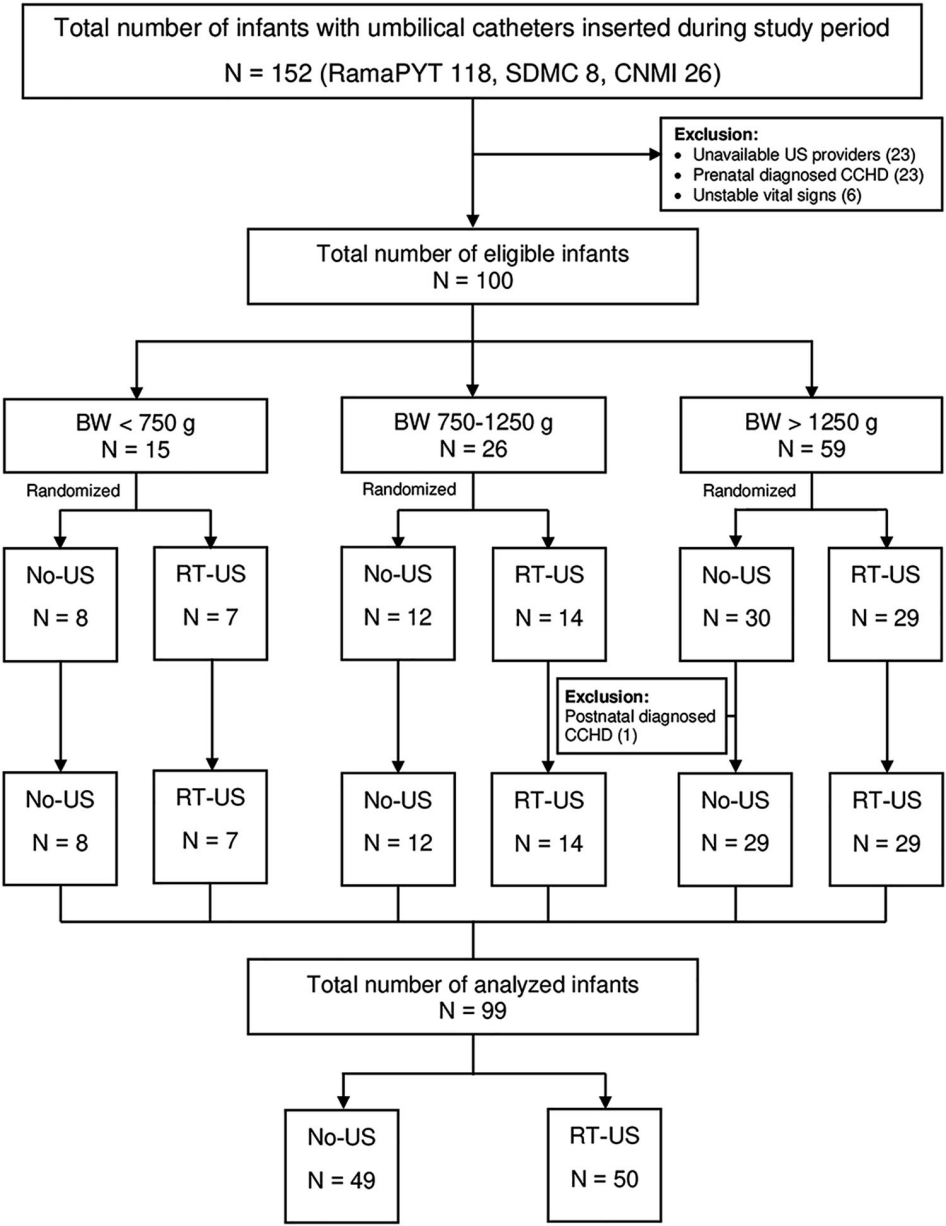
CONSORT diagram.

During the study period, 179 neonates from 3 NICUs required umbilical catheter placement on admission. Seventy-nine neonates were excluded; therefore, 100 neonates were enrolled into the study. Following randomization, one neonate in the No-US group was further excluded, leaving 49 neonates in the No-US group and 50 neonates in the RT-US group for analysis (Fig. 1).

There were no significant differences in the neonates’ baseline characteristics between the two groups. In the No-US group, 79 umbilical catheters (49 UVCs and 30 UACs) were placed, whereas in the RT-US group, 87 umbilical catheters (50 UVCs and 37 UACs) were placed. There was no significant difference in the number of umbilical catheters (UVCs and UACs) placed in neonates in the overall cohort or in each birth weight strata between the two groups (Table 1).

**Table 1:** Baseline characteristics.

	All *N* = 99	No-US *N* = 49	RT-US *N* = 50	*p* value
Patient characteristics				
Gestational age, weeks^b^	31.77 ± 4.1	32.31 ± 4.4	31.25 ± 3.8	0.20
Study site *n* (%)				
• RamaPYT	77 (77.7)	38 (77.5)	39 (78.0)	0.99
• SDMC	2 (2.1)	1 (2.0)	1 (2.0)	
• CNMI	20 (20.2)	10 (20.4)	10 (20.0)	
Birth weight (g)a	1610.6 (980, 1880)	1460.0 (1050, 2560)	1387.5 (975, 1740)	0.78
<750 g, *n* (%)	15 (15.1)	8 (16.3)	7 (14.0)	0.90
750–1250 g, *n* (%)	26 (26.3)	12 (24.5)	14 (28.0)	
>1250 g, *n* (%)	58 (58.6)	29 (59.2)	29 (58.0)	
Small for gestational age, *n* (%)	21 (21.2)	10 (20.4)	11 (22.0)	0.21
Length (cm)^b^	38.8 ± 5.8	39.5 ± 6.4	38.1 ± 5.2	0.24
Males, *n* (%)	56 (56.6)	30 (61.2)	26 (52.0)	0.35
Umbilical catheters				
Lines, *n*	166	79	87	–
UAC, *n* (%)	67 (40.4)	30 (38.0)	37 (42.5)	–
UVC, *n* (%)	99 (59.6)	49 (62.0)	50 (57.5)	–

^a^Median (IQR).

^b^Mean (±SD).

At the first X-ray, the RT-US group had a significantly higher rate of a correct UVC and UAC position than the No-US group (*p* < 0.001). The number of catheter adjustments required after the first X-ray was significantly lower in the RT-US group than in the No-US group (*p* < 0.001). There was a significantly higher number of catheters placed in a low position in the No-US group than the RT-US group (UVC, *p* = 0.026; UAC, *p* = 0.036). There was no significant difference in the number of catheters that were removed between the two groups. The reason for catheter placement in a low position or catheter removal was because of unsuccessful attempts to direct the catheter in the optimal (high) position. However, the decision to leave the catheter in a low position or to remove the catheter was left to the attending physician caring for the neonate.

The total number of X-rays required for the procedure was significantly lower in the RT-US group than in the No-US group (*p* = 0.011). The median radiation dosage per patient was lower in the RT-US group than in the No-US group, but the difference was not statistically significant. The cost of an X-ray per patient in the RT-US group was significantly lower than that in the No-US group (*p* = 0.007).

The average time to perform an US to access the tip of the catheter position was 8.72 ± 3.6 minutes. However, the total time to perform the procedure was similar between both groups. There was no significant difference in the rate of complications between the two groups. One neonate in the RT-US group had group B *Streptococcus* in blood culture collected from a UAC on admission (Table 2).

**Table 2:** Study results.

	No-US (*N* = 79 lines)		RT-US (*N* = 87 lines)		Relative risk (95% CI)	*p* value
	UVC *n* = 49	UAC *n* = 30	UVC *n* = 50	UAC *n* = 37		
Primary outcome						
Proper line position in 1^st^ X-ray, *n* (%)	20 (25.3)		63 (72.4)		*–*	<0.001
Proper line position in 1^st^ X-ray, *n* (%)	10 (20.4)	10 (33.3)	32 (64.0)	31 (83.8)	–	<0.001^a, b^
Improper line position in 1^st^ X-ray, *n* (%)	39 (79.6)	20 (66.7)	18 (36.0)	6 (16.2)	0.45 (0.30–0.67)^e^	
○ High line placement	31	16	11	5	0.24 (0.11–0.53)^f^	<0.001^a, b^
○ Low line placement	8	4	7	1		
Secondary outcome						
Low line placement, *n* (%)	9 (18.4)	1 (3.3)	1 (2.0)	0 (0)	*–*	0.026^a^, 0.036^b^
Lines needing removal, *n* (%)	0 (0)	3 (10.0)	1 (2.0)	0 (0)	*–*	0.5^a^, 0.085^b^
Number of line adjustment^c^	1 (1, 2)	1 (0, 1)	0 (0, 1)	0 (0, 0)	*–*	<0.001^a, b^
Number of line adjustment, time per line	1.05		0.29		*–*	<0.001
Number of line adjustment, time per line	1.18	0.83	0.38	0.16	*–*	<0.001^a, b^
Number of line adjustment, *n*						
0 time	10	10	32	31		<0.001
1 time	26	15	17	6	0.32 (0.19-0.54)^e^	
2 times	9	5	1	0	0.19 (0.08-0.47)^f^	
3 times	2	0	0	0		
4 times	2	0	0	0		
Number of X-rays^c^, time per patient	2 (1, 2)		1 (1, 2)		–	<0.001
Total Number of X-rays, films	90		65		–	0.011
Number of X-rays for each patient						
1 X-ray	22		36			
2 X-rays	16		13		0.71 (0.51–0.97)	0.034
3 X-rays	8		1			
4 X-rays	3		0			
Total radiation exposure per patient, dGy*cm^2c^	0.24 (0.08, 0.40)		0.15 (0.10, 0.23)		–	0.192
Cost of X-ray per patient, USD^d^	41.04 ± 13.76		36.98 ± 11.73		–	0.007
Final X-ray confirmation for proper placement, number of patient (%)	29/49 (59.2)		43/50 (86.0)		–	0.01
Duration for US performance, min	–		8.72 ± 3.6		–	–
X-ray waiting time, min^c^	15 (12, 30)		20 (15, 30)		–	0.933
Total procedure time, min^d^	46.94 ± 16.03		43.74 ± 16.05		–	0.192
Complications, number of patient (%)						
Hypothermia	2 (4.1)		1 (2.0)			
Hypoxia	0		4 (8.0)			
Hypotension	0		0		–	0.376
Brady-tachycardia	0		0			
Central line infection/sepsis	0		1 (2.0)			

^a^p of UVC.

^b^p of UAC.

^c^Median (IQR).

^d^Mean (± SD).

^e^Relative risk of UVC.

^f^Relative risk of UAC.

The inter-rater agreement between neonatal fellows, neonatal staff (CR), and pediatric radiologist (NR) in determining the accuracy of the catheter position on X-ray and US was high in all eligible neonates, with k values of 0.84 and 0.81, respectively. The operators’ final agreement remained consistent, regardless of the neonate’s weight, gestational age, or catheter type.

## Discussion

In our study, the use of RT-US to assess the position of the tip of umbilical catheters resulted in a significant increase in the accuracy of proper UVC and UAC placement. We also found a decreased requirement for catheter adjustment after the first X-ray, and a decreased requirement for a follow-up X-ray image after the catheter adjustment.

To the best of our knowledge, our study was one of the few RCT to demonstrate that RT-US improves the accuracy of umbilical catheter placement. Our findings are consistent with a small RCT by Fleming et al. that compared US-guided UVC and UAC insertion with the conventional approach (calculation based on anthropometric measurements) [10]. Although they did not find a difference in the number of UAC manipulations between the two groups, US-guided insertion decreased the number of UVC manipulations, time of catheter placement, and number of X-rays taken. Kaur et al. conducted a similar RCT, but only UVC placement was investigated with similar results to the other trial [11].

In our study, the accuracy of UVC placement significantly enhanced from 20.4% to 64.0% when RT-US was used instead of Shukla’s formula. Previous investigations reported an accuracy of Shukla’s formula ranging from 17.8% to 57.0% for UVC insertion [9, 11, 15–19]. The variation in accuracy could be attributed to different definitions of the optimal catheter position (e.g., inferior vena cava to right atrial junction, 0.5–1.0 cm above the diaphragm or at the 8–10th thoracic vertebrae). Our results were consistent with those previously report in the literature. Rossi et al. performed a retrospective cohort study and found improved accuracy of UVC placement from 46.2% to 58.8% when US was used instead of Shukla’s formula [9]. Additionally, Kaur et al. observed improved accuracy of UVC placement from 26.0% to 57.7% when US was used instead of typical formula-based insertion [11]. Similarly, the accuracy of UAC placement increased considerably from 33.3% to 83.8% when RT-US was used instead of Shukla’s formula. Previous investigations reported an accuracy of Shukla’s formula ranging from 52.4% to 90.6% for UAC insertion [15, 16, 18–20]. However, research on US-guided UAC placement is scarce. In the study of Rossi et al., only two neonates received US for UAC placement, and no analysis of UAC was performed [9].

In this study, we did not intend to use US to help mobilize the catheter through the inferior vena cava passing through the liver and ductus venosus. However, a recent study reported that US can be used for this purpose. In 2024, Misha et al. compared the failure rate of US-guided UVC insertion with the blind approach and found no significant difference between the two groups (20.6% vs. 29.6%, *p* = 0.27) [12]. However, when catheter insertion failed in the blind group, US-guided reinsertion was attempted, and this helped route the catheter into the proper position in 30% of cases. This possibility could explain the decreased rate of low catheter placement and fewer catheters removed in the RT-US group in our study.

The results of multiple investigations, including our own, indicate that the methods currently used for calculating the length of umbilical catheter insertion are not accurate [9, 11, 15–20] Therefore, we developed an equation (linear regression) using the data from our study on the optimum catheter length (not including the umbilical stump length) in relation to the neonate’s birth weight to predict the length of umbilical catheters. The equations for the UVC and UAC are as follows: UVC length (cm) = birth weight (kg) + 5.26 (R^2^ = 0.63, *p* < 0.001) and UAC length (cm) = 2 × birth weight (kg) + 9.60 (R^2^ = 0.79, *p* < 0.001). Interestingly, a previous study in northern Thailand that aimed to determine a new formula to calculate the UVC length using data of 135 Thai neonates reported closely comparable equation to ours [21].

In our study, the total number of catheter readjustments was considerably lower in the RT-US group than in the No-US group (RR: 0.32, 95% CI 0.19-0.54, *p* < 0.001 in UVC; RR 0.19, 95% CI 0.08-0.47, *p* < 0.001 in UAC). Our findings are consistent with a previous study of Fleming et al. who found that the number of UVC manipulations was lower in patients who had US than in those with standard methods (2.8 to 1.6 times per patient, *p* = 0.002) [10]. However, in their study, the number of UAC manipulations was not significantly different between the two methods.

In our study, the total number of X-rays performed in the RT-US group was significantly lower than that in the No-US group (RR 0.71, 95% CI 0.51–0.97, *p* = 0.034). A study by Fleming et al. found a reduction in the number of X-rays per patient from 4.1 in the control group to 2.3 in the US group [10]. Rossi et al. reported that the number of X-rays per patient was lower from 1.5 to 1.19 after US was used to assist in catheter placement [9]. Rubortone et al. showed a reduction in the number of X-rays performed in pre/post intervention when US was used to replace X-rays for confirming the UVC tip position (92.3% vs. 32.1%, *p* < 0.001) [22]. Kaur et al. also reported a 45% reduction in additional X-ray exposure in the group that used US-guided UVC insertion [11]. In our study, there was a trend toward lower radiation exposure in the RT-US group than in the No-US group, however this was not statistically significant. In contrast, Rossi et al. found that the effective radiation dosage (mSv) per episode was significantly lower in the US group [9].

The average X-ray expense per episode was considerably higher in the No-US group than in the RT-US group because of a reduction in the number of X-rays performed in each patient. However, the expense of an US examination was not included in this study. Rossi et al. also showed that US used was cost-effective with a cost saving of £19.33 per line [9].

In this study, the decision to perform a repeated X-ray after catheter adjustment was left to the physician who performed the procedure. Some physicians may not want to repeat X-rays following catheter manipulation, while others might demand them to verify catheter insertion in the proper position. Although the latter might be preferable from a medico-legal standpoint, the final X-ray confirmation of proper placement was performed only in 52.9% of neonates in the No-US group and in 86.0% of neonates in the RT-US group. If final X-ray confirmation of proper catheter placement became mandatory, there would be a considerably higher disparity in the number of X-rays obtained, X-ray expense, and possibly radiation exposure between the two groups.

In the RT-US group, the average time to perform an US to access the tip of the catheter position was approximately 8 minutes, which is surprisingly similar to that reported in the study of Mishra et al. in 2024 [12]. In our NICUs, the routine procedure is to contact the radiology service and use a portable digital X-ray machine to verify the correct positioning of the UVC and UAC after successfully placing them in vessels with an expected insertion length and adequate blood flow. This practice provides an interval (varying from 10 to 45 minutes) in which the RT-US procedure can be performed to accurately locate and adjust the catheter before the X-ray technician’s arrival. Consequently, the use of RT-US had no effect on the total duration of the procedure or the workflow. In contrast, neonates in the RT-US group were more likely to have a suitable catheter location confirmed in the initial X-ray. This finding could account for the trend toward a slightly reduced total procedure duration in the RT-US group. Several authors reported that US-guided UVC placement reduces the total procedure duration because it decreases the requirement for repeated adjustment or eliminates the requirement for an X-ray [11, 12, 22]. Increasing evidence suggests that US may be more accurate than X-rays in determining the position of umbilical catheters. Therefore, if US had been the primary method utilized to evaluate the placement in our study, the overall procedure time may have been reduced even further.

Our study used AP chest X-ray as the gold standard for determining the umbilical catheter tip position because it is the standard of care in our NICUs and the most commonly used modality to confirm tip placement worldwide [3, 4]. However, the optimum method to determine the umbilical catheter position is subject to debate. This optimum method is controversial because, in studies where US was performed on an appropriately positioned UVC documented on an X-ray, a large percentage of these catheters were shown to be malpositioned (likely to be too high, e.g., located in the right atrium) [23, 24]. A recent systematic review that compared X-ray with US to verify the UVC tip position reported an X-ray sensitivity of 0.90 (95% CI 0.7-0.97) and a specificity of 0.82 (95% CI 0.53–0.95) [25]. In our study, more than half of the catheters in the RT-US group that were evaluated as being in an improper position and needed to be adjusted after an X-ray were those in which the radiologist was unable to comment on their position in an US image (10/18 in UVCs and 3/6 in UACs). The overall inter-rater reliability (k values) of an US image specifically for these catheters was relatively low (UVC: 0.220; UAC: 0.217). Poor US visualization of the catheter tip is likely to be the main explanation of these findings, emphasizing the importance of the abilities of the provider, which may be addressed by standardized protocols and training [22, 26].

There were no significant differences in short- and long-term complications, such as hypothermia, hypotension, brady-tachycardia, and desaturation, during the procedure between the two groups. Despite the theoretical concern that an US scan may contaminate the sterile field and increase the risk of central line infection or sepsis, there was no significant difference in the infection rate between the two groups in this study. The group B *Streptococcus* in blood culture of a neonate in the RT-US group could have been vertically transmitted. Similar to our study, Mishra et al. identified central line-associated bloodstream infection in 9 of 98 patients in their study [12]. However, there was no difference in these incidents between the US-guided UVC insertion and blind groups.

### Limitations

The limitations of our study include a small sample size, which may lead to an overestimation of results. Additionally, we have limited long-term outcome data and did not evaluate the financial expenses of US usage. The open-label design of the study may have introduced bias, although we implemented several measures to minimize this. All catheters in the controls were strictly inserted according to the standard formula. The definition of a catheter tip’s proper positioning on an X-ray was predetermined and adhered to rigorously. The physician responsible for the catheter insertion decided on the manipulation of the catheter and the necessity of additional X-rays, with the expectation of adhering to high standards of practice. To ensure quality, all X-ray and US images were reviewed by a radiologist (NR) who was not involved in patient care. Lastly, our study was conducted across three university hospital NICUs operated by the same group of physicians with consistent training and practices, which may limit the generalizability of our findings to other hospital settings. Despite that, the results of our study may help in developing comprehensive training programs that promote ongoing education and proficiency, thereby supporting wider adoption and effective utilization of this technology.

Growing evidence worldwide underscores the benefits of US as a point-of-care tool in NICUs [5]. As the new generation of neonatologists gains increasing expertise in US, its application for assessing catheter tip placement in neonates is expected to gain more popularity and could potentially be integrated into routine practice. Future research should confirm our findings and address gaps in knowledge, including implementation and training challenges as well as cost-effectiveness and long-term outcomes. Finally, alternative approaches to facilitate the correct positioning of the catheter should be explored and evaluated in comparison to existing techniques.

## Conclusions

RT-US improves the accuracy of UVC and UAC placement, reducing the requirement for catheter adjustments and the number of X-rays performed.

## Data Availability

The datasets used and/or analyzed during the current study are available from the corresponding author upon reasonable request.
